# Genome-Wide Identification of the *TaBSK* Gene Family and Its Salt-Responsive Expression Patterns in Wheat

**DOI:** 10.3390/cimb48080816

**Published:** 2026-08-12

**Authors:** Yongtao Zhao, Junsen Wang, Zhongzhou Zhang, Qian Yuan, Shicong Zhen, Hao Guo, Chuan Xia, Zhenchen Xie

**Affiliations:** 1 Luohe Academy of Agricultural Sciences, Luohe 462300, China; 2 State Key Laboratory of Crop Gene Resources and Breeding, Institute of Crop Sciences, Chinese Academy of Agricultural Sciences, Beijing 100081, China

**Keywords:** wheat, *TaBSK*, gene family, Brassinosteroids (BRs), salt stress

## Abstract

Brassinosteroid signaling kinases (BSKs) act as core signal transducers downstream of Brassinosteroid (BR) perception and integrate plant growth regulation with broad-spectrum biotic and abiotic stress tolerance. Despite well-established functional characterizations of *BSK* gene families in *Arabidopsis thaliana* and rice, comprehensive genome-wide profiling and salt response analysis of *BSK* homologs remain lacking in wheat. In this study, we systematically identified 18 *TaBSK* family members. Phylogenetic analysis separated wheat *TaBSKs* into three distinct evolutionary subgroups. The 18 *TaBSK* loci were unevenly distributed across 14 chromosomes derived from the A, B, and D subgenomes. Motif scanning uncovered 10 universal conserved amino acid motifs, including two signature functional domains: the tetratricopeptide repeat (TPR) and protein kinase catalytic domain (PKc). Intra-genomic collinearity analysis confirmed that segmental duplication constituted the primary evolutionary driver underlying *TaBSK* family expansion. Extensive *cis*-regulatory element profiling identified abundant hormone- and stress-responsive *cis*-motifs. Transcriptome profiling RNA-seq datasets revealed five *TaBSK* genes exhibiting significant differential transcription under salt stress. Specifically, *TaBSK16*, *TaBSK17*, and *TaBSK18* were markedly upregulated following salt exposure. Collectively, this study delivers an evolutionary and transcriptional atlas of the wheat *TaBSK* family and provides candidate genes for functional validation and molecular breeding toward salt-tolerant wheat varieties. Collectively, this study explores the evolution and transcriptional patterns of the wheat *TaBSK* gene family and provides candidate genes for subsequent functional validation and molecular breeding of salt-tolerant wheat varieties.

## 1. Introduction

Brassinosteroids (BRs) are a class of indispensable steroid phytohormones that widely exist in higher plants and play central regulatory roles in coordinating diverse developmental events and stress adaptation processes throughout the entire plant life cycle [1,2]. Physiologically, BRs participate in the modulation of cell elongation and division, floral transition, seed morphogenesis, and senescence progression, while also enhancing plant resistance against a variety of adverse environmental conditions, thereby balancing plant growth and defense responses [3,4,5].

Brassinosteroid signaling kinases (BSKs) constitute a vital family of cytoplasmic protein kinases and serve as essential intermediate signal transducers in the canonical BR signaling pathway [6]. Upon the perception of BR signals by plasma membrane-located receptor complexes, BSK proteins are activated to relay upstream signaling cascades to downstream transcriptional regulators, thereby triggering the expression of BR-responsive genes and initiating a series of physiological and biochemical responses [7]. Since the first discovery and functional characterization of *BSK* family genes in the model plant *Arabidopsis thaliana*, extensive genetic and physiological evidence has validated the multifaceted biological functions of AtBSK proteins [8,9]. Specifically, *AtBSK* genes not only govern BR-modulated plant growth and morphological development but also positively regulate plant adaptation to diverse biotic and abiotic stresses, including drought, salinity, extreme temperature, and pathogenic microbial infection [10,11,12]. In addition, the homologous *OsBSK* genes in rice have been systematically characterized in recent years. Multiple *OsBSK* family members have been verified to positively regulate grain development, yield component formation, and salt stress acclimation, indicating the conserved and diversified functions of *BSK* genes in governing crop yield and stress resilience [13,14,15,16].

Bread wheat (*Triticum aestivum* L.) is the most widely cultivated staple cereal worldwide, supplying approximately 20% of total human caloric and protein intake, and sustaining international food security [17,18]. Nevertheless, continuous soil salinization caused by irrational irrigation, coastal intrusion, and desertification has become a predominant abiotic stress factor that severely restricts sustainable wheat production across the globe [19,20,21]. Excessive salt accumulation in soil disrupts cellular ion homeostasis, triggers severe osmotic stress and reactive oxygen species (ROS) burst, damages cell membrane integrity, inhibits photosynthetic efficiency and root development, and ultimately leads to stunted plant growth and a reduced spike number and grain weight [22,23]. In major saline cultivation areas, salt stress can cause a wheat yield reduction of 60%, which poses a serious threat to regional agricultural stability and grain supply [24]. Therefore, deciphering the molecular regulatory mechanisms of wheat salt tolerance and excavating core stress-responsive genes is critical for the genetic improvement of salt-tolerant wheat varieties.

Although the regulatory roles of *BSK* family genes in plant growth and stress responses have been well documented in *Arabidopsis thaliana* and rice, these findings cannot be directly extrapolated to wheat due to the distinct genomic complexity and genetic specificity of allohexaploid wheat. Previous studies on *Arabidopsis thaliana* (diploid) and rice (diploid) focused on simple, single-subgenome *BSK* gene families with fewer homologous copies and straightforward genetic regulatory networks, which fail to reveal the functional differentiation, subgenome bias, and expression specificity of *BSK* genes in polyploid crops [25,26]. Bread wheat possesses a complex AABBDD hexaploid genome with an enormous genome size of ~17 Gb, abundant repetitive sequences and massive homoeologous gene copies derived from three distinct progenitor subgenomes [27,28]. This unique polyploid genomic characteristic leads to widespread functional redundancy, neofunctionalization, and the subfunctionalization of wheat homoeologous genes, resulting in distinct gene regulatory patterns and stress response mechanisms compared with diploid model plants. These genomic features also substantially hinder comprehensive gene family identification, comparative structural analysis and in vivo functional verification [29,30]. More importantly, the tissue-specific expression patterns, temporal salt response dynamics and underlying transcriptional regulatory mechanisms of wheat *TaBSK* paralogs under salt stress remain largely unelucidated, and high-value salt-tolerant candidate *TaBSK* genes have not been systematically screened and validated. The lack of comprehensive research on wheat *BSK* family members limits our understanding of BR-mediated salt tolerance mechanisms in wheat and restricts the application of *BSK* genetic resources in wheat salt tolerance improvement.

Given the conserved crucial roles of *BSK* genes in plant stress adaptation and the urgent agricultural demand for improving salt tolerance in wheat germplasm, a comprehensive genome-wide characterization and transcriptional profiling of the *TaBSK* gene family are of great theoretical and practical significance. In this study, we systematically characterized all 18 *TaBSK* family members and analyzed their physicochemical properties, phylogenetic evolutionary relationships, conserved gene structures, chromosomal distribution patterns, and *cis*-acting regulatory elements. Combined with RNA-seq transcriptomic datasets and quantitative RT-PCR (RT-qPCR) experimental validation, we further explored the dynamic temporal expression profiles of *TaBSK* genes under salt stress and screened salt-responsive candidate genes. Our findings fill the current research gap in our understanding of the wheat *BSK* gene family, and deliver valuable genetic resources for the molecular breeding of high-yield and salt-tolerant wheat varieties.

## 2. Materials and Methods

### 2.1. Genome-Wide Mining and Physicochemical Parameter Calculation of TaBSK Members

Genomic assembly data, protein sequences, and gene annotation GFF3 files of the reference wheat cultivar Chinese Spring (IWGSC RefSeq v2.0) were retrieved from the Ensembl Plants database (https://ftp.ebi.ac.uk/pub/ensemblorganisms/GCA/900/519/105/1/community_iwgsc/2018_04/, accessed on 10 May 2025). Full-length BSK protein reference sequences of *Arabidopsis thaliana* and japonica rice were separately downloaded from the TAIR database (https://www.arabidopsis.org/, accessed on 10 May 2025) and Rice Genome Annotation Project (https://rice.uga.edu/, accessed on 10 May 2025) (Supplementary Table S1), respectively.

Two complementary screening strategies were integrated to capture all putative TaBSK homologs: (1) BLASTP searches were conducted using AtBSK and OsBSK protein sequences as queries against wheat proteome with E-value ≤ 1 × 10^−5^ in TBtools-II (version 2.313) [31]. (2) Hidden Markov Model (HMM) profiles of PKc (PF07714) and TPR (PF07719) were retrieved from the Pfam database (http://pfam.xfam.org/, accessed on 10 May 2025) [32]. HMM search was carried out against the bread wheat proteome with an E-value cutoff of 1 × 10^−5^. All candidate protein sequences retrieved from dual screening pipelines were further validated through InterPro (https://www.ebi.ac.uk/interpro/, accessed on 12 May 2025) conserved domain verification to retain only sequences harboring both canonical BSK signature domains. Final confirmed *TaBSK* genes were functionally annotated via the Triticeae-GeneTribe platform (http://wheat.cau.edu.cn/TGT/, accessed on 12 May 2025) [33].

Basic biochemical parameters of TaBSK proteins, including total amino acid count, molecular weight (MW), theoretical isoelectric point (pI), instability index, aliphatic index, and grand average of hydropathicity (GRAVY), were computed using the built-in Protein Parameter Calculator module in TBtools-II (version 2.313). Subcellular localization predictions were performed using two independent online predictive tools: DeepLoc 2.1 (https://services.healthtech.dtu.dk/services/DeepLoc-2.1/, accessed on 15 May 2025) and CELLO (http://cello.life.nctu.edu.tw/, accessed on 15 May 2025), with consistent localization results adopted for subsequent analysis.

### 2.2. Cross-Species Phylogenetic Reconstruction of BSK Homologs

Full-length amino acid sequences of all identified TaBSKs, AtBSKs, and OsBSKs were subjected to multiple sequence alignment using the ClustalW alignment with default parameter settings in MEGA 12 (version 12.0.11) software [34]. A neighbor-joining (NJ) evolutionary tree was constructed with 1000 bootstrap replicates for branch confidence assessment, Poisson amino acid substitution correction, and pairwise gap deletion treatment. Raw phylogenetic tree output was imported into the iTOL online visualization platform (http://itol.embl.de/; accessed on 26 May 2025) for aesthetic refinement and taxonomic clade color annotation [35].

### 2.3. Conserved Motif, Functional Domain and Gene Structural Analysis

Conserved protein motifs across all TaBSK paralogs were identified using the MEME Suite online toolkit (https://meme-suite.org/meme/tools/meme; accessed on 28 May 2025) [36]. The maximum detectable motif number was set to 10, while all other search parameters remained default settings. Conserved functional domains of each TaBSK protein were reconfirmed via InterPro (https://www.ebi.ac.uk/interpro/; accessed on 28 May 2025) database scanning.

Exon–intron structural features and untranslated region (UTR) coordinates were extracted by mapping *TaBSK* gene coding sequences to Chinese Spring genome GFF3 annotations. TBtools-II (version 2.313) Gene Structure View (Advanced) module was utilized to visualize gene architectures. Integrated composite figures combining phylogenetic tree topology, conserved motif distribution, domain architecture, and exon–intron arrangements were generated for holistic comparative analysis of all *TaBSK* members.

### 2.4. Chromosomal Mapping, Gene Duplication and Collinearity Analysis of TaBSK Members

Chromosomal positional information of each *TaBSK* locus was extracted from genome annotation GFF3 files and visualized via TBtools-II (version 2.313) chromosomal localization plotting tools. Intra-genomic duplication events within the *TaBSK* family were detected using MCScanX algorithms embedded in TBtools-II (version 2.313), with syntenic duplication pairs illustrated through the Advanced Circos visualization module.

### 2.5. Promoter Cis-Regulatory Element Profiling

For each *TaBSK* gene, 2000 bp upstream genomic sequences extending from the transcription start site were extracted from the Chinese Spring reference genome. *Cis*-acting regulatory elements (CAREs) within these promoter fragments were annotated by batch querying the PlantCARE database (https://bioinformatics.psb.ugent.be/webtools/plantcare/html/; accessed on 30 May 2025) [37]. Annotated *cis*-motifs were categorized into three major functional groups: plant growth and development-related elements, hormone-responsive elements, and biotic/abiotic stress-inducible elements. The positional distribution of all detected *cis*-regulatory motifs across *TaBSK* promoters was visualized as a combined heatmap-style diagram in TBtools-II (version 2.313).

### 2.6. Salt Stress Treatment and RNA-Seq Transcriptome Sequencing

The salt-tolerant wheat cultivar DeKang 961 (DK961) was selected for salt stress transcriptome analysis. Sterilized DK961 seeds were germinated and hydroponically cultured in standardized nutrient solution containing 27.218 mg/L KH_2_PO_4_, 120.36 mg/L MgSO_4_, 111.825 mg/L KCl, 166.47 mg/L CaCl_2_, 0.6183 mg/L H_3_BO_3_, 0.0618 mg/L (NH_4_)_6_Mo_7_O_24_•4H_2_O, 0.1248 mg/L CuSO_4_•5H_2_O, 0.2875 mg/L ZnSO_4_•7H_2_O, 0.1690 mg/L MnSO_4_•H_2_O, 36.705 mg/L FeNaEDTA, and 472.30 mg/L Ca(NO_3_)_2_•4H_2_O. Solution pH was stabilized at 6.0 via HCl or NaOH adjustment, and full nutrient replacement was performed every 72 h. Two-week-old seedlings with three fully expanded leaves were subjected to continuous NaCl treatment for 6 h, 12 h, and 24 h. All plant materials were cultivated in a controlled phytotron with a 16 h light/8 h dark photoperiod, constant temperature (25 °C daytime/18 °C nighttime), and ~70% relative humidity.

At each sampling time point, roots from three seedlings were pooled to form one independent biological replicate. Three such replicates were collected for transcriptome sequencing. Total RNA was extracted using a modified CTAB-PBIOZOL extraction protocol coupled with ethanol precipitation purification. RNA concentration and purity were quantified using a Qubit 4.0 fluorometer (Thermo Fisher Scientific, Waltham, MA, USA), while RNA integrity was assessed via a Qsep400 capillary fragment analyzer (Advanced Instruments, USA Advanced Instruments, Inc., Norwood, MA, USA). RNA library construction and Illumina sequencing services were commissioned to Tsingke Biotechnology Co., Ltd. (Tianjin, China).

Raw sequencing reads were preprocessed with fastp (version 0.24.0) to remove low-quality bases, adapter sequences, and short contaminated reads [38]. Clean high-quality reads were aligned against the IWGSC RefSeq v2.0 wheat reference genome using HISAT2 (version 2.1.0) software [39]. Raw read count matrices were imported into DESeq2 for differential expression statistical analysis (Supplementary Table S2) [40]. FPKM (fragments per kilobase of transcript per million mapped fragments) values were calculated to normalize transcript abundance for cross-sample expression comparison. Genes satisfying FDR < 0.05 and |log_2_(Fold Change)| ≥ 1 thresholds were defined as significantly differentially expressed genes (DEGs) under salt stress.

### 2.7. Total RNA Extraction and RT-qPCR Expression Validation

Parallel salt stress treatment was replicated on two-week-old DK961 seedlings for RT-qPCR verification at 6 h, 12 h, and 24 h post-NaCl exposure. Seedling root total RNA was isolated using TRIzol reagent (Invitrogen, Carlsbad, CA, USA) following manufacturer operating instructions. RNA absorbance values at OD_260_/OD_280_ were detected via NanoDrop spectrophotometry to evaluate purity, and 1.2% agarose gel electrophoresis was performed to confirm intact RNA bands. Full-length first-strand cDNA reverse transcription was conducted using the 5×All-In-One RT Master Mix kit (Abm Inc., New York, NY, USA). Quantitative real-time PCR amplification was carried out on a LIGHTCYCLER 96 real-time fluorescence quantitative instrument (Roche, Basel, Switzerland). Three independent RNA extraction batches served as biological replicates, with three technical replicates amplified per sample. The wheat *TaActin* gene was selected as the internal reference gene for expression normalization. Relative expression was calculated using the 2^−ΔΔCT^ comparative threshold cycle method. Data are presented as mean ± SD with three biological replicates. Significant differences among groups were analyzed by one-way ANOVA coupled with Tukey’s test, and lowercase letters indicate differences at *p* < 0.05. All target gene-specific primer sequences are listed in Supplementary Table S3.

## 3. Results

### 3.1. Identification and Physicochemical Characterization of TaBSK Family Proteins

After integrating BLASTP homology screening, HMM domain scanning, and InterPro conserved domain validation, a total of 18 *TaBSK* genes were identified from the Chinese Spring genome. Complete physicochemical parameters of all TaBSK proteins were summarized in Table 1. Substantial variation in protein structural parameters was observed across family members, while subcellular localization predictions exhibited complete conservation across all paralogs.

The amino acid length of TaBSK proteins ranged from 482 residues (TaBSK18) to 527 residues (TaBSK6), with an average length of ~508 amino acids. Corresponding molecular weights spanned 53,828.83 Da (TaBSK15) to 58,728.77 Da (TaBSK6), reflecting moderate size divergence within the gene family. The theoretical pI values varied from 5.28 (TaBSK7/9) to 6.30 (TaBSK4), and 10 out of 18 TaBSK members possessed pI values between 5.81 and 6.26. The protein instability indices ranged from 35.47 (TaBSK5) to 50.41 (TaBSK2). By standard biochemical classification, proteins with instability index > 40 are classified as intracellularly unstable. In total, 11 TaBSK paralogs were predicted unstable, while the remaining seven (TaBSK1/3/5/12/14/15/18) exhibited high intracellular stability (instability index < 40). The aliphatic index of TaBSK proteins ranged from 68.45 (TaBSK13) to 84.00 (TaBSK12), with an average value of 76.3. All 18 TaBSK proteins had negative GRAVY values (−0.513 to −0.196). Subcellular localization prediction by two independent software tools consistently indicated that all TaBSK proteins were localized to the plasma membrane. Collectively, the *TaBSK* gene family displays broad biochemical divergence in protein size, charge properties, and intracellular stability.

### 3.2. Cross-Species Phylogenetic Relationships of BSK Homologs

**Figure 1:**
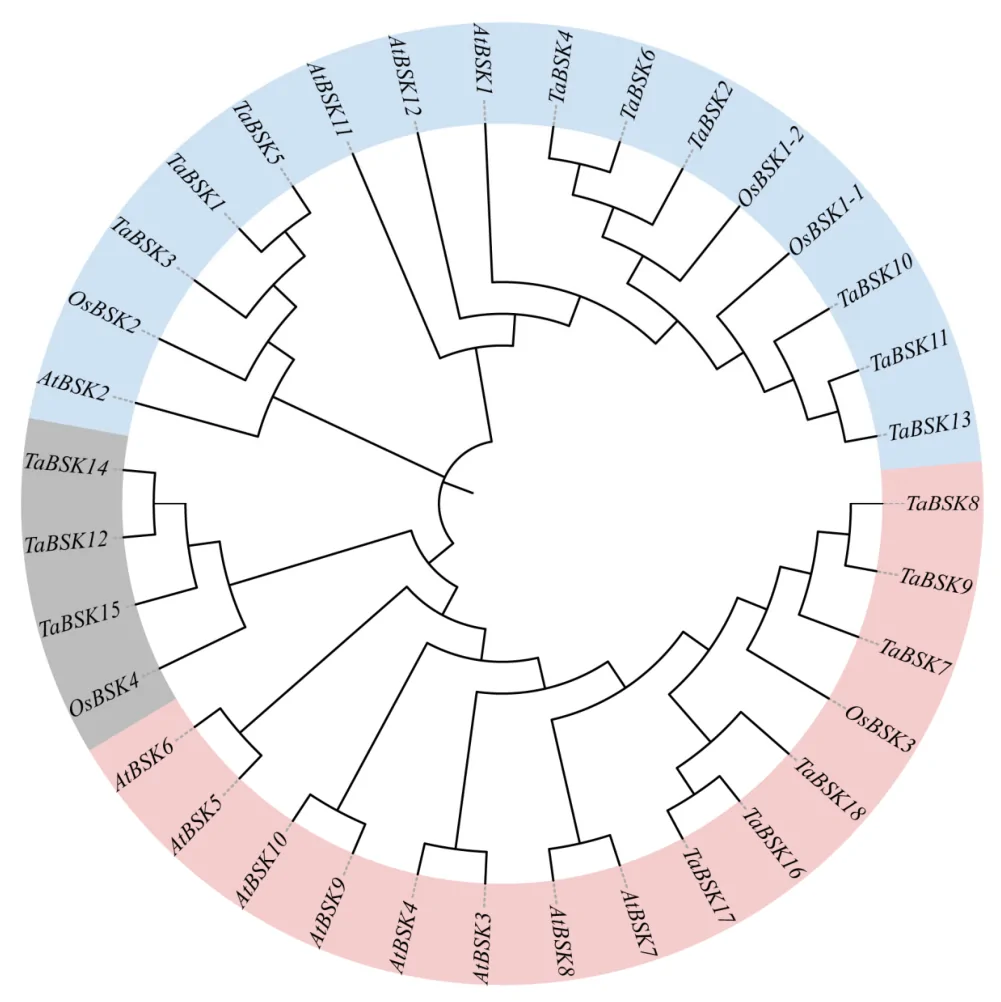
Circular neighbor-joining phylogenetic tree of BSK proteins from wheat (TaBSK), rice (OsBSK), and *Arabidopsis thaliana* (AtBSK). Branch coloration distinguishes taxonomic groups to visualize cross-species orthology and lineage-specific gene expansion.

A total of 18 TaBSK proteins from wheat, OsBSK paralogs from rice, and AtBSK members from *Arabidopsis thaliana* were aligned to construct a circular neighbor-joining phylogenetic tree to explore the evolutionary relationships of the *BSK* gene family across monocot and dicot species (Figure 1). The tree clearly divided all BSK proteins into three distinct, well-supported monophyletic clades distinguished by different background color shading: the blue monocot clade I, the gray monocot clade II, and the pink dicot-specific clade III. Clade III (pink region) exclusively contained AtBSK proteins derived from the dicot model plant *Arabidopsis thaliana*, forming an independent lineage separated from grass BSK homologs. Clades I and II consisted of monocot BSK proteins originating from wheat and rice. Clade I accommodated the majority of TaBSK members (TaBSK1/2/3/4/5/6/10/11/13) alongside multiple rice OsBSK paralogs (OsBSK1-1/1-2/2). Clade II harbored TaBSK12/14/15 together with OsBSK4, forming a small conserved monocot subclade. Notably, several wheat-specific sub-branches were observed within the two monocot clades, including the clustered TaBSK8/9 pair and the TaBSK16/17/18 triad in the pink-edged monocot sub-branch adjacent to the dicot clade. In addition, each homoeologous gene triplet of *TaBSKs* originating from the A, B and D subgenomes of wheat clustered tightly on adjacent branches of the tree.

### 3.3. Conserved Motif, Functional Domain and Exon–Intron Structural Analysis of TaBSKs

**Figure 2:**
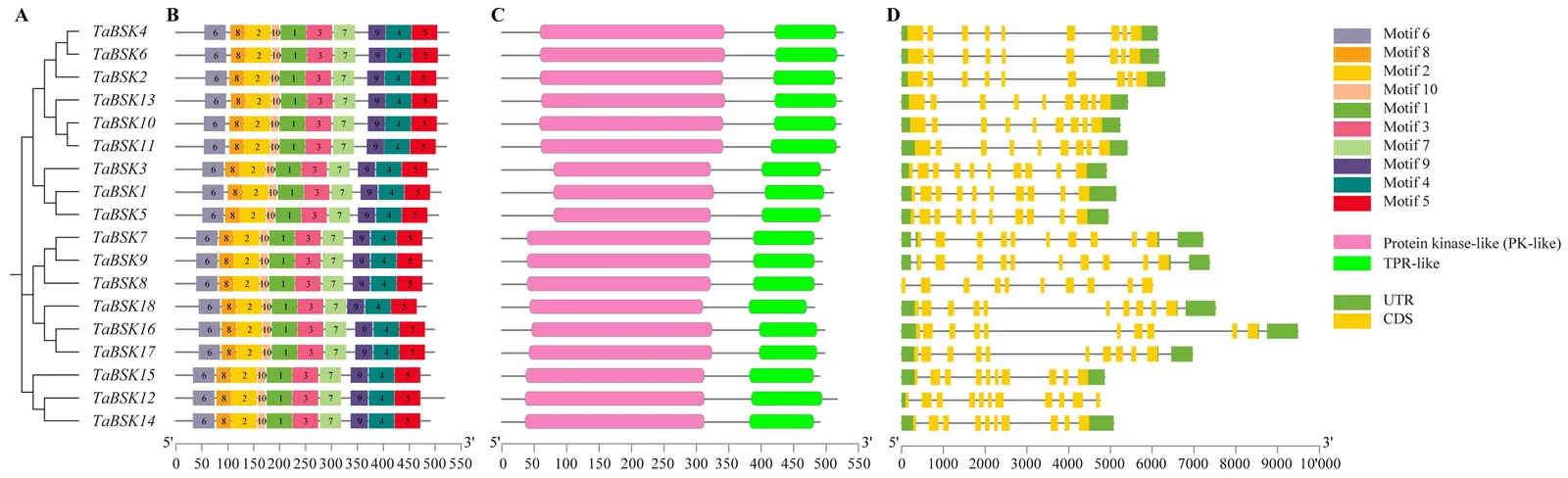
Integrated structural analysis of wheat *TaBSK* gene family members. (**A**) Intra-family phylogenetic tree. (**B**) Color-coded distribution of 10 conserved MEME-derived amino acid motifs. (**C**) Schematic diagram of conserved domain architecture. (**D**) Gene structure. A unified scale bar normalizes relative sequence lengths across all panels.

Intra-family phylogenetic analysis, MEME motif prediction, InterPro domain annotation, and exon–intron structure analysis were performed for all 18 *TaBSK* genes (Figure 2). A separate phylogenetic tree was first generated solely for all 18 TaBSK protein sequences to resolve intra-family evolutionary relationships (Figure 2A). MEME motif scanning identified 10 conserved protein motifs (Motif 1–10) across all TaBSK paralogs (Figure 2B). Paralogous genes within the same phylogenetic subclade exhibited highly consistent motif composition. InterPro domain scanning verified two non-negotiable signature domains present in every TaBSK protein: an N-terminal PKc protein kinase catalytic domain and a C-terminal TPR tetratricopeptide repeat domain (Figure 2C). Exon–intron structural mapping revealed substantial variation in total gene length (1000 bp to 9000 bp), yet paralogs within identical phylogenetic clades exhibited highly comparable intron–exon arrangements (Figure 2D). Overall, integrated multi-layer structural analysis demonstrates that the wheat *TaBSK* gene family retained core functional domains and clade-specific gene architectures.

Gene structural analysis revealed obvious variations in total gene length (1000–9000 bp) among *TaBSK* members, while genes clustered in the same phylogenetic clade displayed highly similar intron–exon arrangement patterns.

### 3.4. Chromosomal Localization and Synteny of TaBSK Loci

**Figure 3:**
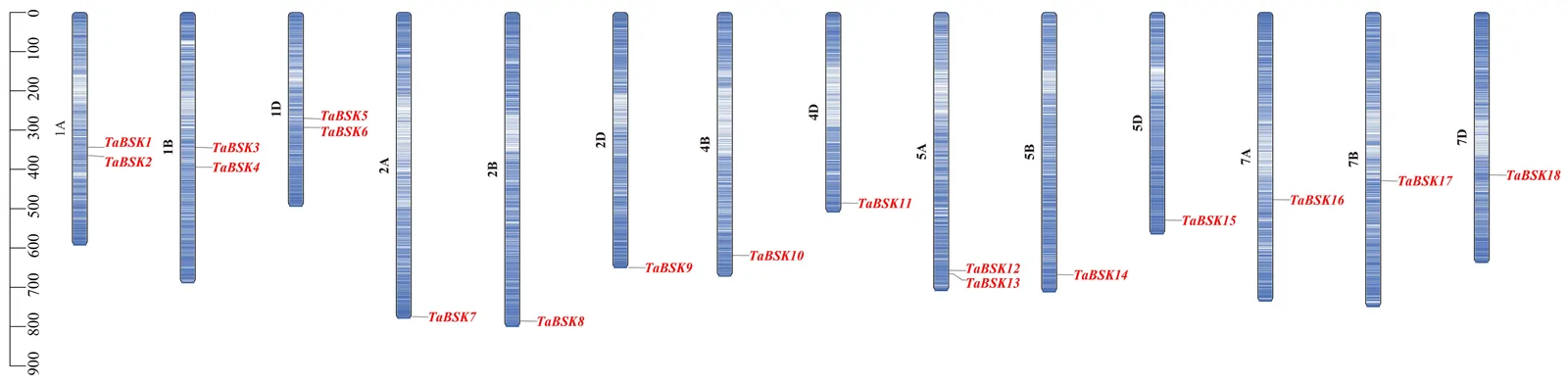
Chromosomal localization map of all 18 *TaBSK* genes in wheat. Vertical blue bars represent individual chromosomes labeled by homoeologous group and subgenome (A/B/D). Red text labels denote *TaBSK* gene names positioned at their physical loci. Left-side scale bars indicate relative chromosome length in megabases (Mb).

Chromosomal positional mapping demonstrated uneven, non-random distribution of the 18 *TaBSK* loci across 14 chromosomes derived from wheat’s A, B, and D subgenomes (Figure 3). Homoeologous group 1 chromosomes each carry two *TaBSK* paralogs: *TaBSK1/2* on 1A, *TaBSK3/4* on 1B, and *TaBSK5/6* on 1D. Homoeologous group 2 chromosomes contain discrete gene arrangements: single loci *TaBSK7* (2A), *TaBSK8* (2B), and *TaBSK9* (2D). Chromosome 4B and 4D harbors a solitary *TaBSK10* (4B) and *TaBSK11* (4D) locus. Group 5 chromosomes exhibit two paralogs on 5A (*TaBSK12*, *TaBSK13*) and single loci on 5B (*TaBSK14*) and 5D (*TaBSK15*). Isolated single *TaBSK* genes were assigned to 6A (*TaBSK16*), 7A (*TaBSK17*), and 7D (*TaBSK18*). This asymmetric chromosomal distribution pattern suggests differential duplication and retention events shaped *TaBSK* family expansion during wheat polyploid evolution.

**Figure 4:**
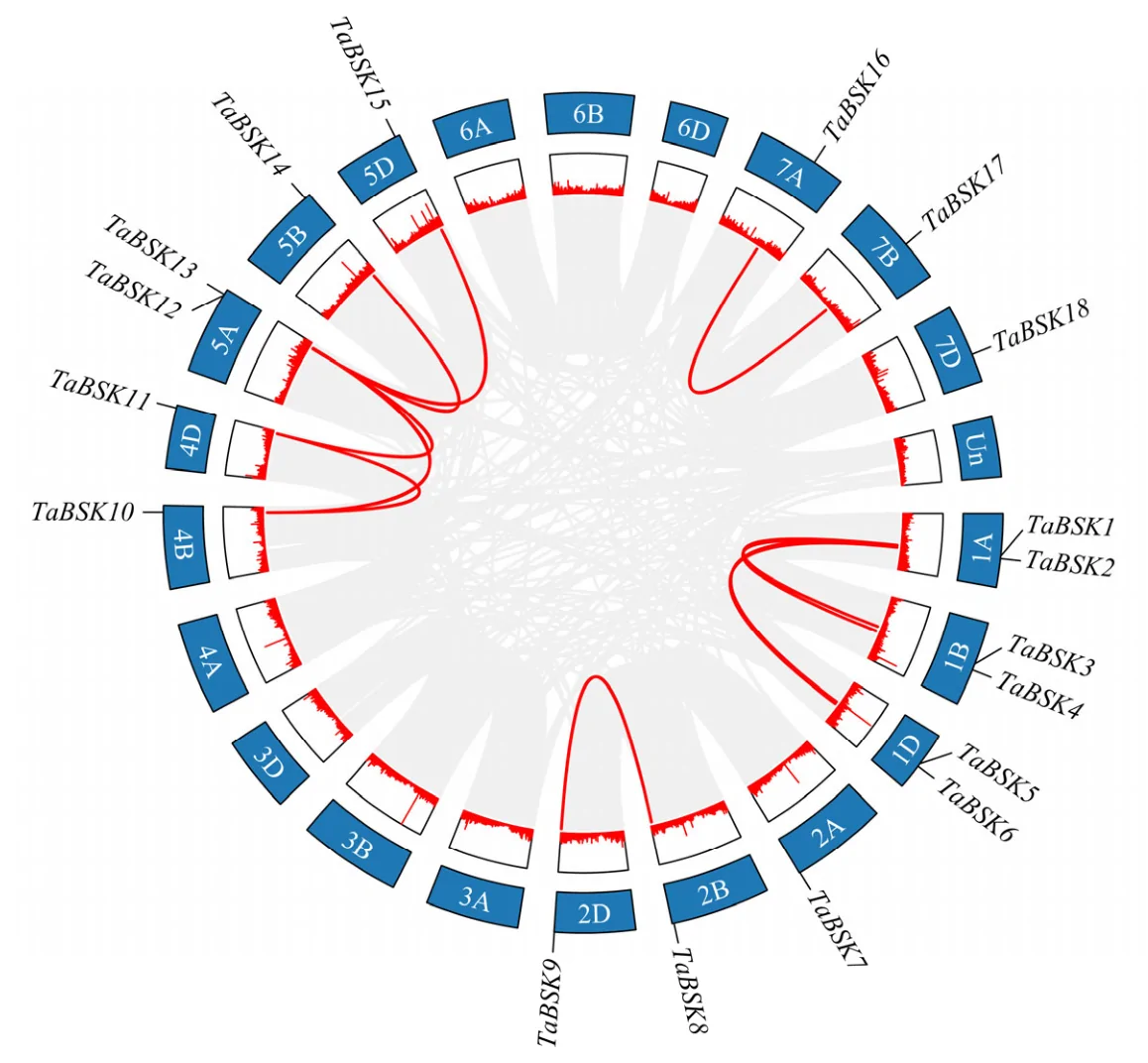
Genome-wide collinearity and duplication analysis of the *TaBSK* gene family. Chromosomes are displayed as rectangular blue tracks annotated with subgenome identifiers. Red arcs connect collinear duplicated *TaBSK* gene pairs; dense grey background lines illustrate global inter-chromosomal syntenic blocks across the wheat genome. The “Un” label designates unanchored genomic scaffolds lacking mapped *TaBSK* loci.

Intra-genomic synteny analysis visualized via Circos plots confirmed extensive collinear connections between homoeologous *TaBSK* gene pairs across A/B/D subgenomes (Figure 4). Clear collinear pairing was detected among group 1, 4 and 5 homoeologous *TaBSK* triads, confirming segmental duplication and polyploid homoeolog retention as the dominant evolutionary forces driving *TaBSK* gene family expansion. All homoeologous *TaBSK* triads displayed balanced retention across corresponding A, B, and D subgenome chromosomes, indicating no severe subgenomic bias against *TaBSK* paralogs during hexaploid wheat stabilization.

### 3.5. Cis-Regulatory Element Diversity Within TaBSK Promoter Regions

**Figure 5:**
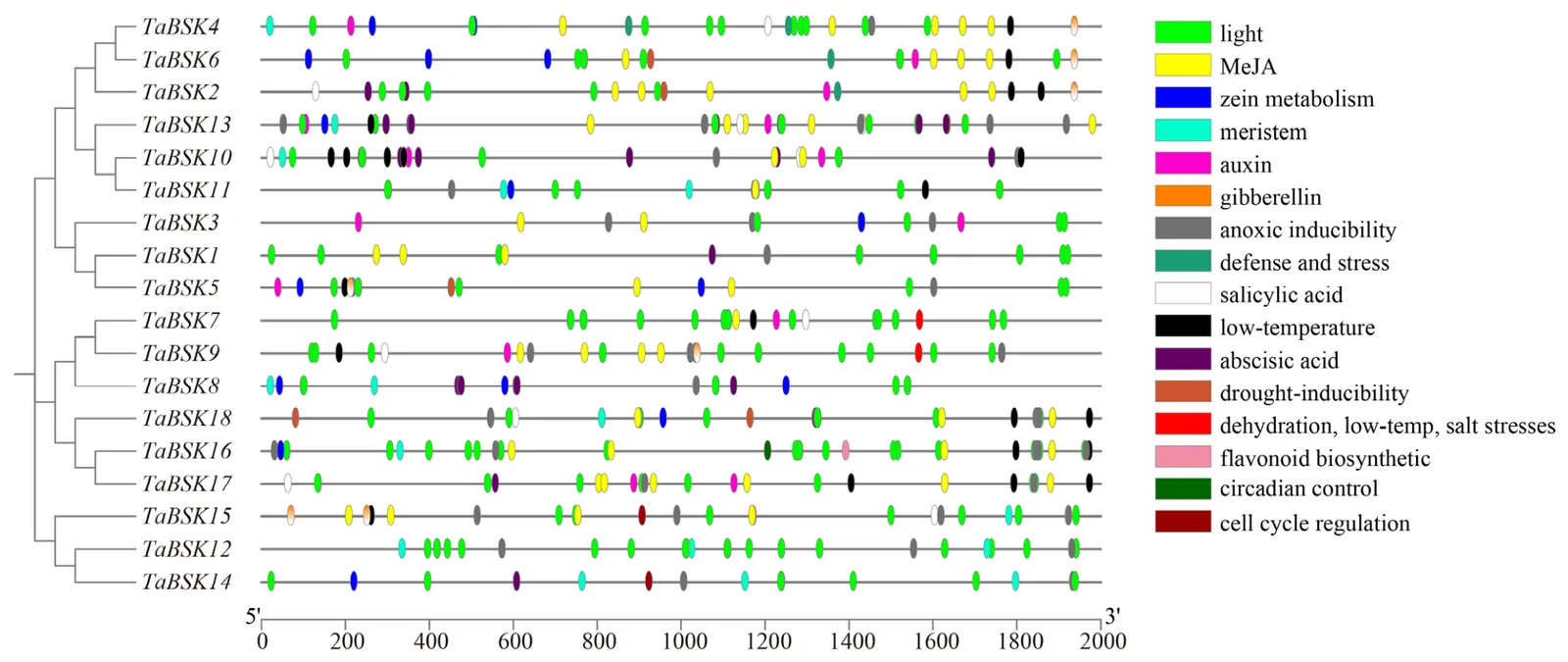
Distribution of *cis*-acting regulatory elements across the 2000 bp promoter sequences of all *TaBSK* genes. The left vertical tree represents intra-family TaBSK phylogenetic relationships. Each horizontal black line corresponds to a full-length promoter fragment, with colored dots marking the exact base-pair position of distinct functional *cis*-motifs.

Comprehensive *cis*-element screening was performed on the 2000 bp upstream promoter sequences of every *TaBSK* gene to decode transcriptional regulatory inputs governing *TaBSK* expression (Figure 5). Hundreds of distinct *cis*-motifs were detected, functionally categorized into light-responsive, hormone-signaling, abiotic/biotic stress-responsive, and developmental regulatory elements. Light-associated *cis*-motifs constituted the most abundant and universally distributed elements, present in all 18 *TaBSK* promoter sequences. Multiple hormone-responsive *cis*-elements were widely distributed across the gene family, such as Methyl jasmonate (MeJA), ABA, gibberellin, auxin, and salicylic acid responsive motifs. A rich repertoire of stress-inducible *cis*-regulatory motifs was also annotated, supporting *TaBSK* functionality during adverse environmental adaptation. Low-temperature responsive motifs, drought-inducible responsive elements, and defense-related motifs were broadly distributed across *TaBSK* promoters. The DRE/CRT dehydration/salt/low-temperature responsive *cis*-motifs were identified in multiple *TaBSK* upstream regions, directly linking *TaBSK* transcriptional regulation to salt stress signaling. Development-associated *cis*-elements were also recovered, including circadian rhythm regulatory motifs, cell cycle elements, and zein metabolism motifs, indicating *TaBSK* contributions to diurnal expression cycling and grain developmental processes.

Notably, the quantity, positional arrangement, and subtype composition of *cis*-regulatory motifs varied drastically between individual *TaBSK* paralogs. This promoter heterogeneity drives transcriptional divergence within the gene family, enabling separate *TaBSK* paralogs to specialize in distinct hormone signaling, stress response, and developmental regulatory pathways.

### 3.6. Time-Dependent Transcriptional Shifts in TaBSK Genes Under Salt Stress

**Figure 6:**
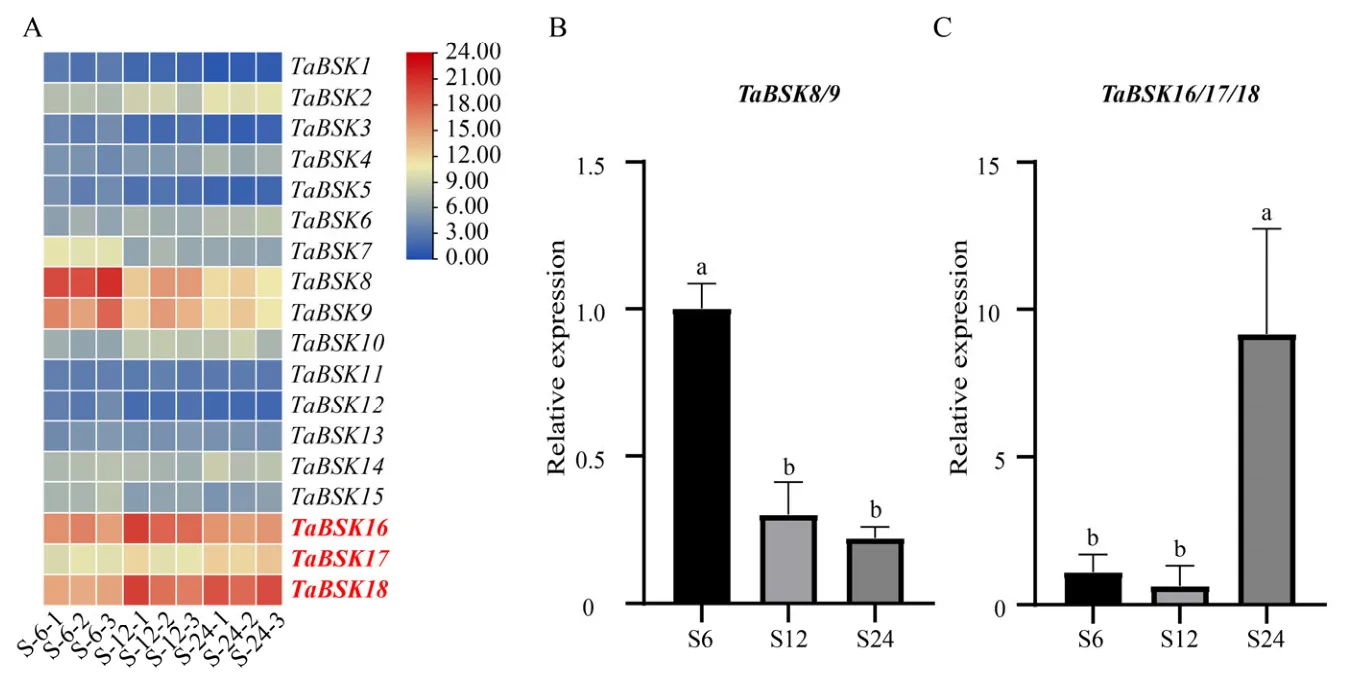
Time-resolved transcriptional profiling of *TaBSK* genes under 6 h, 12 h, and 24 h salt stress treatment. (**A**) Global transcript abundance heatmap of all 18 *TaBSK* paralogs based on RNA-seq FPKM values. Color intensity correlates with relative expression magnitude. Two subgroups (*TaBSK8/9*, *TaBSK16/17/18*) display prominent stage-specific upregulation. (**B**) RT-qPCR relative expression of *TaBSK8/9*. (**C**) RT-qPCR quantification of the *TaBSK16/17/18* subgroup. Columns bearing distinct lowercase letters indicate statistically significant expression differences (*p* < 0.05). Error bars denote SEM of biological replicates.

RNA-seq transcriptome heatmaps revealed differential expression patterns of all 18 *TaBSK* paralogs at three salt treatment time points (6 h, 12 h, 24 h post-NaCl exposure; abbreviated S6, S12, S24) (Figure 6A). Most *TaBSK* genes (*TaBSK1–7*, *TaBSK10–15*) maintained weak to moderate transcript accumulation across all three sampling time points. Two discrete paralog subgroups exhibited time-specific transcriptional activation. The *TaBSK8/9* clade peaked at S6, while the *TaBSK16/17/18* subgroup displayed robust transcriptional induction exclusively at S24, suggesting temporally partitioned physiological functions for these two clades during salt acclimation.

RT-qPCR quantification validated the distinct temporal expression trajectories of these two high-abundance subgroups (Figure 6B,C). Combined relative expression of *TaBSK8* and *TaBSK9* reached maximum levels at S6, followed by significant transcriptional repression at S12 and S24, with no statistical difference observed between S12 and S24 expression values. The *TaBSK16/17/18* subgroup’s exhibited basal expression remained low at S6 and S12, while transcript levels surged to nearly 9-fold relative expression at S24. Collectively, transcriptional profiling demonstrates that major high-expression *TaBSK* clades operate at discrete salt stress response.

## 4. Discussion

BSKs act as core cytoplasmic signal integrators in the canonical BR signaling cascade, linking plasma membrane BR receptors complexes and downstream modules to coordinate plant vegetative development, reproductive growth, and broad-spectrum tolerance to biotic and abiotic stressors across all angiosperm lineages [41,42].

### 4.1. Filling the Knowledge Gap of BSK Gene Family in Hexaploid Wheat

To date, systematic genome-wide explorations of *BSK* gene families and their expression patterns have been characterized in multiple diploid model plants and cash crops, such as *Arabidopsis thaliana* [8], rice [13], cotton [43], spinach [44] and Alfalfa [45], while comparable multi-dimensional characterization in wheat remained a critical unaddressed knowledge gap prior to the present study. Polyploid species possess complex gene duplication, subgenome differentiation, and functional redundancy characteristics, making it impossible to directly extrapolate the regulatory rules of diploid plant *BSK* genes to wheat. In this study, we systematically identified 18 non-redundant canonical *TaBSK* paralogs at the genome-wide level, and comprehensively analyzed their protein properties, evolutionary relationships, gene structures, *cis*-regulatory elements, and salt-induced temporal expression patterns. This study provides evolutionary and transcriptional insights into the wheat *TaBSK* family, enriches the current understanding of BR signalling gene families in polyploid cereals, and identifies candidate genes relevant to studies on wheat salt-tolerance molecular breeding.

### 4.2. Structural Conservation, Evolutionary Divergence and Regulatory Plasticity of TaBSK Gene Family

Gene family evolution is dominated by two complementary strategies: functional conservation maintaining core biological pathways and sequence divergence driving adaptive differentiation. Our structural analysis revealed that all 18 TaBSK proteins harbor the typical N-terminal PKc kinase domain and C-terminal TPR domain and consistent plasma membrane localization, which is consistent with the conserved structural characteristics of *BSK* family genes in *Arabidopsis thaliana* and rice. The PKc domain undertakes core phosphorylation catalytic functions, while TPR domains mediate protein interactions with the BRI1-BAK1 receptor complex, and universal plasma membrane localization further confirms the retention of canonical BR signaling functions during wheat polyploidization [46,47]. Despite conserved domain architecture, TaBSK paralogs exhibit distinct differences in protein length, molecular weight, stability and hydrophilicity. Stable paralogs may maintain basal BR signaling for normal growth, while unstable isoforms are likely involved in rapid signal turnover under acute salt stress, indicating functional sub-specialization of duplicated *TaBSK* genes.

Phylogenetic analysis divided plant BSKs into three monophyletic clades with clear monocot–dicot differentiation, demonstrating that *BSK* duplication events occurred before and after the monocot–dicot divergence. Dicot-specific *AtBSKs* formed an independent clade, while wheat and rice BSKs clustered in monocot-exclusive branches, verifying the conserved ancestral BSK repertoire in grasses. Wheat-specific subclades such as salt-responsive *TaBSK8/9* and *TaBSK16/17/18* further reflect lineage-specific gene expansion during wheat polyploidization. Conserved motif and gene structure analyses corroborated phylogenetic classification, with core functional domains highly conserved and sequence variations mainly limited to flexible linker regions, indicating strong purifying selection on functional domains.

Promoter *cis*-element analysis revealed that the expression of *TaBSK* genes is regulated by the crosstalk of multiple hormone and stress signaling pathways. The widespread presence of light-responsive *cis*-motifs in *TaBSK* promoter sequences reveals the extensive crosstalk between BR signaling and light signal pathways in wheat [48,49]. Meanwhile, the abundant enrichment of *cis*-elements responsive to abscisic acid (ABA), gibberellin (GA), auxin (IAA), jasmonic acid (JA), and salicylic acid (SA) demonstrates that *TaBSK* genes serve as critical integrators that link BR signaling to diverse hormone signaling networks in wheat [50]. The distinct composition and distribution patterns of *cis*-elements among *TaBSK* paralogs lead to divergent spatiotemporal expression patterns, enabling different *TaBSK* members to participate in specific hormone crosstalk, environmental adaptation, and developmental regulation processes, which greatly improves the environmental adaptability of polyploid wheat.

### 4.3. Temporal Functional Partitioning of TaBSK Paralogs in Salt Stress Adaptation

Salt stress is a dynamic progressive process, including early ionic shock and late sustained osmotic stress damage. Plants need to activate staged regulatory networks to cope with different stress stages, but the temporal functional differentiation of wheat salt-tolerant genes has rarely been reported. In this study, time-series transcriptomics and RT-qPCR validation identified a novel two-stage salt regulatory mode of *TaBSK* family in wheat seedlings. The early-responsive *TaBSK8/9* clade was rapidly induced at 6 h of salt stress. In contrast, the late-activated *TaBSK16/17/18* clade was specifically upregulated at 24 h, dominating long-term osmotic adaptation and growth reprogramming. This temporal partitioning strategy facilitates salt stress adaptation in wheat. The candidate genes derived from the salt-tolerant wheat cultivar DK961 provide valuable genetic resources for improving salt tolerance in saline farmland, which severely restricts global wheat yield. Collectively, our multi-omics findings systematically elucidate the evolutionary conservation, functional divergence and salt regulatory patterns of the *TaBSK* family, laying a foundation for exploring BR-mediated salt tolerance mechanisms and molecular breeding in wheat.

### 4.4. Limitations and Future Perspectives

Although this study provides comprehensive bioinformatic and transcriptomic evidence for *TaBSK* salt regulatory functions, several limitations remain to be addressed via in vivo functional verification. First, all current conclusions are based on predictive analysis and expression data, lacking genetic evidence from stable transgenic overexpression and CRISPR-Cas9 knockout wheat lines. Future studies will generate transgenic materials of five key salt-responsive *TaBSK* genes and evaluate their salt tolerance phenotypes, including seedling survival, root growth, Na^+^/K^+^ ratio, osmolyte content and antioxidant enzyme activity, to confirm their in planta functions. Second, the downstream interacting proteins and phosphorylation targets of TaBSK kinases in BR–salt crosstalk networks remain unclear, requiring further exploration to clarify the molecular mechanism of TaBSK-mediated ion homeostasis and ROS detoxification. Third, the tissue-specific expression patterns of *TaBSK* genes in leaves, stems and developing grains are uncharacterized, limiting our understanding of their roles in seedling salt adaptation and yield stability under field saline stress. In addition, the salt-responsive expression analysis was conducted only in the salt-tolerant cultivar DeKang961. A comparative transcriptomic analysis between salt-tolerant and salt-susceptible wheat cultivars will be necessary in future studies to elucidate the differential roles of *TaBSK* genes in salt-stress responses and to identify candidate genes for molecular breeding. Further functional validation will transform our descriptive gene-family atlas into mechanistic insights, bridging the gap between genome-wide screening and practical wheat salt-tolerance breeding.

## 5. Conclusions

This study systematically identified and characterized 18 *TaBSK* gene family members in wheat. These paralogs were unevenly distributed on 14 chromosomes of the A, B, and D subgenomes, and segmental duplication contributes to the expansion of this gene family under purifying selection. All TaBSK proteins retain canonical PKc kinase and TPR interaction domains, with universal plasma membrane subcellular localization. Numerous *cis*-elements associated with light, hormone and abiotic stress responses were identified in *TaBSK* promoter regions. Transcriptome and RT-qPCR analyses revealed distinct temporal expression patterns of *TaBSK* genes under salt stress, with *TaBSK8/9* activated during early salt exposure and *TaBSK16/17/18* induced at late stress stages. The findings provide valuable molecular information and candidate genes for further functional characterization of *TaBSK* genes and salt-tolerance breeding in wheat.

## Figures and Tables

**Table 1:** Physicochemical parameters and predicted subcellular localization of 18 TaBSK proteins in wheat.

Gene Name	AA (aa)	MW (Da)	pI	Instability Index	Aliphatic Index	GRAVY	SL
*TaBSK1*	511	56,330.14	5.89	35.83	82.56	−0.34	Cell membrane
*TaBSK2*	524	58,571.49	6.26	50.41	70.86	−0.51	Cell membrane
*TaBSK3*	506	55,903.71	5.89	36.05	82.79	−0.334	Cell membrane
*TaBSK4*	526	58,556.44	6.3	49.68	70.59	−0.509	Cell membrane
*TaBSK5*	506	55,807.54	5.89	35.47	82.61	−0.332	Cell membrane
*TaBSK6*	527	58,728.77	6.26	49.19	71.56	−0.492	Cell membrane
*TaBSK7*	494	55,428.75	5.28	44.48	75.12	−0.419	Cell membrane
*TaBSK8*	494	55,370.71	5.34	44.17	75.12	−0.413	Cell membrane
*TaBSK9*	494	55,428.75	5.28	44.48	75.12	−0.419	Cell membrane
*TaBSK10*	523	58,302.01	6.05	48.96	68.59	−0.513	Cell membrane
*TaBSK11*	521	57,921.72	6.26	47.36	69.42	−0.472	Cell membrane
*TaBSK12*	517	57,098.8	5.81	37.4	84	−0.196	Cell membrane
*TaBSK13*	524	58,431.28	6.19	49.43	68.45	−0.511	Cell membrane
*TaBSK14*	490	53,872.93	5.83	35.8	81.49	−0.271	Cell membrane
*TaBSK15*	490	53,828.83	5.75	36.1	81.69	−0.265	Cell membrane
*TaBSK16*	498	56,163.79	5.45	40.58	77.07	−0.387	Cell membrane
*TaBSK17*	498	56,227.92	5.53	41.51	77.27	−0.384	Cell membrane
*TaBSK18*	482	54,464.81	5.51	39.84	74.59	−0.412	Cell membrane

Abbreviations: AA (aa), Amino Acid Residues; MW (Da), Molecular Weight (Daltons); pI, Theoretical Isoelectronic Point; GRAVY, Grand Average of Hydropathicity; SL, Subcellular Localization.

## Data Availability

The original contributions presented in this study are included in the article/Supplementary Material. Further inquiries can be directed to the corresponding author.

## Supplementary Materials

The following supporting information can be downloaded at: https://www.mdpi.com/article/10.3390/cimb48080816/s1.
